# Balancing Closeness and Detachment in Family Dementia Caregiving: A Mixed Methods Review

**DOI:** 10.1093/geroni/igaf122.2916

**Published:** 2025-12-31

**Authors:** Josey Batura, Gavin Green, Heather Kelly, Elizabeth Fauth

**Affiliations:** Utah State University, Logan, Utah, United States; Utah State University, Logan, Utah, United States; Institute for Disability Research, Policy & Practice, Logan, Utah, United States; Utah State University, Logan, Utah, United States

## Abstract

**Background:**

Caring for a family member with Alzheimer’s disease and related dementias (ADRD) involves significant relational shifts, including changes in reciprocity, emotional closeness, and relational strain. While caregiver burden is well-documented, less is known about how caregivers perceive and adapt to changes in relationship closeness and whether closeness is uniformly beneficial.

**Objectives:**

This mixed methods systematic review examines (1) how family caregivers experience changes in relationship closeness, (2) the psychosocial factors that predict closening versus detachment, and (3) the impact of perceived closeness on caregiver well-being and caregiving outcomes.

**Methods:**

A systematic search was conducted following PRISMA guidelines across PubMed, EBSCOhost, Scopus, and ProQuest. Inclusion criteria required empirical, peer-reviewed studies published since 2015 focusing on relational closeness in spousal ADRD caregiving. A total of 15 studies met the criteria.

**Results:**

Three themes emerged: (1) shifting relational roles, (2) emotional adaptation, and (3) the bidirectional impact of closeness on caregiver and care recipient well-being. Some caregivers experienced deepened bonds, particularly when they maintained a sense of partnership despite role transitions. Others experienced detachment, often as a psychological buffer against the emotional distress of declining reciprocity. Closeness was typically linked to lower burden and greater resilience, but also greater anticipatory grief and distress.

**Conclusion:**

Closeness is not universally beneficial; while it can foster resilience, it may also intensify grief and distress. Future research should explore longitudinal trajectories of closeness and assess whether interventions (e.g., dyadic counseling, structured grief support) can help caregivers balance emotional connection with adaptive coping strategies.

